# Review of Medicinal Plants Traditionally Used to Treat Diarrhea by the People in the Amhara Region of Ethiopia

**DOI:** 10.1155/2023/8173543

**Published:** 2023-11-25

**Authors:** Destaw Damtie

**Affiliations:** Department of Biology, College of Sciences, Bahir Dar University, Bahir Dar, Ethiopia

## Abstract

**Background:**

Diarrheal illness is the second-most common cause of death in under-five children. Worldwide, it results in about 1.7 billion illnesses and 525,000 deaths among under-five children annually. It is the leading cause of malnutrition among under-five children. Different people use medicinal plants to treat diarrhea. The present study aimed to review the medicinal plants used to treat diarrhea by the people in the Amhara region and to diagnose whether the antidiarrheal activities of the medicinal plants have been confirmed by studies using animal models.

**Methods:**

The author searched 21 articles from worldwide databases up to December 2022 using Boolean operators (“AND” and “OR”) and the terms “ethnobotanical studies,” “ethnobiology,” “traditional medicine,” “ethnobotanical knowledge,” and “Amhara region.”

**Results:**

From the 21 studies reviewed, 50 plant species grouped into 28 families were reported to treat diarrhea by the people in the Amhara region. The top most used families were *Lamiaceae* (12%), *Fabaceae* (8%), *Asteraceae, Cucurbitaceae, Euphorbiaceae*, and *Poaceae* (6% each). The modes of administration of the plant parts were orally 98.88% and topically 1.12%. The different extracts of 18 (or 36%) of the medicinal plants traditionally used to treat diarrhea by the people in the Amhara region have been proven experimentally in animal models.

**Conclusions:**

The people in the Amhara region use different medicinal plants to treat diarrhea. Most of them take the medicinal plants orally. The traditional claim that 60% of medicinal plants are antidiarrheal has been confirmed in *in vitro* studies.

## 1. Background

Diarrhea is the second leading cause of under-five mortality in the world [1]. In 2019 alone, diarrheal diseases resulted in 6.58 billion incident cases, 99 million prevalent cases, 1.53 million deaths, and 80.9 million disability-adjusted life years (DALYs) [2]. Among under-five children, diarrheal diseases resulted in 45.5 million DALYs and 370,000 deaths in 2019 [1, 2]. There are three clinical types of diarrhea, namely, acute watery diarrhea, which may last several hours or days and includes cholera; acute bloody diarrhea, also called dysentery; and persistent diarrhea, lasting 14 days or longer [1].

The Amhara region in Ethiopia experiences varying rates of diarrhea prevalence among under-five children, as indicated by several studies. A systematic review and meta-analysis published in PLOS ONE found that the overall prevalence of diarrhea in the region was 21%, which closely aligns with the national prevalence of 22% [3]. However, individual studies conducted in specific areas within the Amhara region showed different prevalence rates. For example, studies in Bahir Dar city reported a prevalence of 14.5% [4], while Farta district showed a higher prevalence of 29.9% among under-five children [5]. Other areas such as Jawi district, Debre Berhan town, Woldia town, Bahir Dar Zuria district, the rural area of the North Gondar zone, and flood-prone villages of the Fogera and Libo Kemkem districts also exhibited varying prevalence rates ranging from 15.5% to 29.0% [6–10]. Interestingly, a report suggested that there was no significant variation in prevalence between high and low hotspot districts in the region [11]. By integrating the wisdom and methodologies of traditional and modern medicine, a comprehensive and holistic healthcare approach can be established to prevent and treat diarrheal diseases in the Amhara region. This collaborative approach has the potential to improve the overall effectiveness of the healthcare system and advance the well-being of the local population.

Herbal medicines are believed to be effective in curing diarrhea, and for many years, plants and plant extracts have been used to treat various gastrointestinal ailments, including diarrhea [12, 13]. However, herbal medicines used in the treatment of diarrhea in African rural communities are unlikely to be replaced soon by modern medicines [14].

Nowadays, the integration of herbal medicine into modern medical practices is highly advocated [15]. Furthermore, herbal medicines have active components that serve as prototype leader compounds for the development of new drugs [16]. Documenting herbal medicines is thus documenting future drugs. Its ecological and cultural diversity make Ethiopia a rich source of herbal medicine [17]. However, due to environmental degradation, deforestation, a lack of recordkeeping, and potential acculturation, the plants and related indigenous knowledge in the nation are steadily diminishing [18]. Therefore, documentation of traditional knowledge regarding the usage of medicinal herbs is crucial to ensure its use by both present and future generations [19]. Hence, the present study aims to document medicinal plants from the Amhara region of Ethiopia that is traditionally used to treat diarrhea.

## 2. Research Methodology

### 2.1. Purpose

Documentation of medicinal plants of antidiarrheal importance is essential for local knowledge conservation, formulating antidiarrheal drugs from plant extracts, and the isolation of interesting compounds to synthesize future effective antidiarrheal drugs.

### 2.2. Search Strategy

The author searched articles from PubMed/Medline, Science Direct, Web of Science, and Google Scholar up to December 2022 by using Boolean operators (“AND” and “OR”) and the terms “diarrhea,” “dysentery,” “ethnobotanical studies,” “ethnobiology,” “traditional medicine,” “ethnobotanical knowledge,” and “Amhara region.”

### 2.3. Eligibility Criteria

The present study included articles written in English and published until December 2022 dealing with the documentation of indigenous knowledge and articles that possess the scientific names, family names, local names, plant parts used, routes of administration, the way of using plants, and the modes of preparation.

### 2.4. Quantitative Analysis of Ethnobotanical Data

Since the study is a review study, the author faced problems searching for data to compute many of the quantitative parameters. Accordingly, only relative frequency of citation (RFC) and family use value (FUV) are found to be applicable to this study. They were calculated using the following formulae:

(1) $$\begin{array}{c} \text{RFC}=\frac{\text{Number of citations to }a\;\text{species}}{\text{Total number of citations to all the species}},\\ \text{FUV}=\frac{\text{The number of species in each family used to treat diarrhea}}{\text{Total number of species in all the families used to treat diarrhea}}. \end{array}$$

## 3. Results

### 3.1. Identification of Relevant Articles

**Figure 1:**
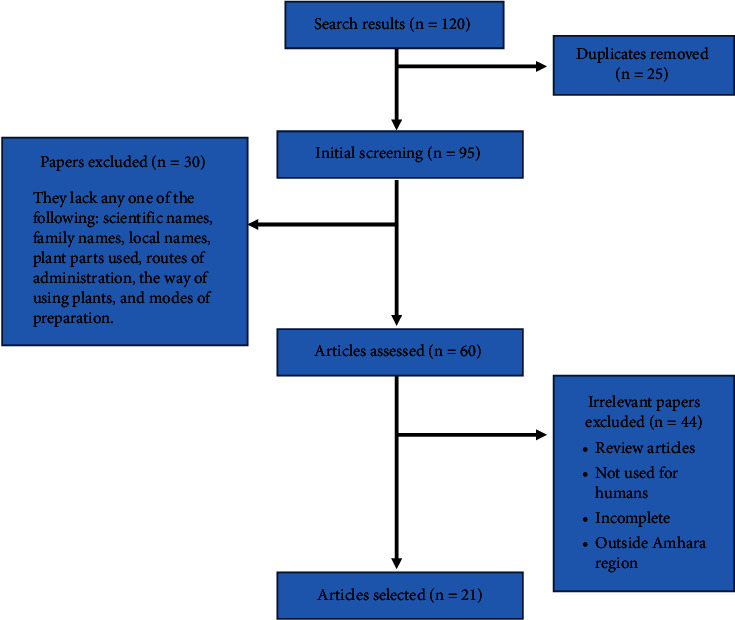
Flowchart of the screening process for this review.

A literature search by the authors turned up a total of 120 published papers. 21 articles were chosen for this review after removing duplicates and irrelevant articles (Figure 1).

### 3.2. List of Identified Plants Used to Treat Diarrhea in the Study Area

From the 21 studies eligible for this study, 50 plant species were reported to treat diarrhea (Table 1). They are *Acacia abyssinica* [32], *Acacia etbaica* Schweinf [31], Aloe spp. [21], *Anogeissus leiocarpa* (A. Rich) Guill. and Perr [23], *Artemisia abyssinica* [24–26], *Balanites aegyptiaca* (L.) Delile [21], *Calpurnia aurea* (Ait). Benth [20, 22, 29, 30, 32, 35, 36], *Carica papaya* L. [28], *Carissa spinarum* L. [26, 29, 30], *Clutia abyssinica* Jaub. and Spach. [22], *Clutia lanceolata* Forssk. [20], *Coffea arabica* L. [20, 29–31, 39], *Cordia africana* [27], *Croton macrostachyus* De [34], *Cucumis ficifolius* [31, 32], *Eragrostis tef.* (Zucc.) Trotter [33], *Ficus thonningii* Blume [34], *Ficus vasta* Forssk [35, 36], *Heteromorpha arborescens* (Spreng). Cham. and Schitdi. [22], *Hordeum vulgare* L. [31, 32], *Justicia schimperiana* (Hochst. ex Nees) T. Anders. [20], *Leonotis ocymifolia* [28, 35, 36]. *Lepidium sativum* L. [26, 28–30], *Linum usitatissimum* L. [22, 24], *Malva parviflora* L. [21], *Mentha piperita* L. [21, 25], *Momordica foetida* Schumach [20], *Myrtus communis* L. [34], *Ocimum lamiifolium* L. [29, 30], *Gossypium barbadense* L. [37], *Plectranthus lactiflorus* (Vatke) Agnew [26], *Prunus persica* (L.) Batsch [20], *Punica granatum* [32], *Rumex abyssinicus* [27], *Rumex nepalensis* (Spreng) [38], *Ruta chalepensis* L. [24, 29, 30], *Salvia nilotica* Jacq. [22], *Satureja punctata* R. Br. [22], *Senna didymobotrya* (Fresen) [31], *Solanecio gigas* (Vatke) C. jeffrey [22], *Solanum nigrum* L. [20], *Sorghum bicolor* (Moench) [22], *Stephania abyssinica* (Dillon and A. Rich.) Walp. [38], *Syzygium guineense* (Willd.) DC. [23], *Verbascum sinaiticum* Benth [20, 34, 38], *Verbena officinalis* L. [22, 26, 28–30, 40], *Vernonia adoensis* Sch.Bip.exWalp. [23], *Withania somnifera* (L.) Dunal [29, 30, 38, 40], *Zehneria scabra* (Linn. f.) Sond [20, 33], and *Ziziphus spina-christi* (L.) Desf [22].

The medicinal plants used by the population in the Amhara region for the treatment of diarrhea are grouped into 28 families and 50 species, as indicated in Table 1. The *Lamiaceae* family was represented by six (12%) species and the *Fabaceae* family by four (8%) species. *Asteraceae*, *Cucurbitaceae*, *Euphorbiaceae*, and *Poaceae* were represented by three (6%) species each. *Caricaceae*, *Malvaceae*, *Moraceae*, *Myrtaceae*, *Polygonaceae*, and *Solanaceae* were represented by two (4%) species each. *Apiaceae*, *Acanthaceae*, *Aloaceae*, *Boraginaceae*, *Brassicaceae*, *Combretaceae*, *Linaceae*, *Menispermaceae*, *Punicaceae*, *Rhamnaceae*, *Rosaceae*, *Rubiaceae*, *Rutaceae*, *Scrophulariaceae*, *Verbenaceae*, and *Zygophyllaceae* were represented by a single (2%) species each. Only two modes of administration of the plant parts were used to treat diarrhea. Almost all (98.88%) were applied orally, and only 1.12% dermally (topically).

### 3.3. Quantitative Analyses of Ethnobotanical Data

#### 3.3.1. Relative Frequency of Citations (RFC)

**Figure 2:**
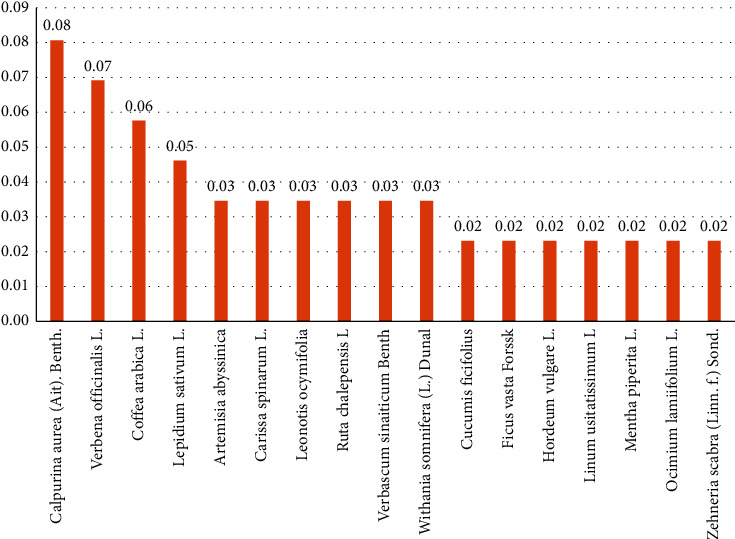
Relative frequency of citations (RFC) of 17 of the 50 species.

The relative frequency of citations (RFC) ranged from 0.01 to 0.08. The top four cited medicinal plants for their antidiarrheal activities were *Calpurnia aurea* (Ait). Benth., *Verbena officinalis* L., *Coffea arabica* L., and *Lepidium sativum* L., with RFC values of 0.08, 0.07, 0.06, and 0.05, respectively. *Artemisia abyssinica*, *Carissa spinarum* L., *Leonotis ocymifolia*, *Ruta chalepensis* L., *Verbascum sinaiticum* Benth, and *Withania somnifera* (L.) Dunal all had the same RFC value of 0.03. Furthermore, 14% and 66% of the species had RFC values of 0.02 and 0.01, respectively (Figure 2).

#### 3.3.2. Family Use Value (FUV)

**Figure 3:**
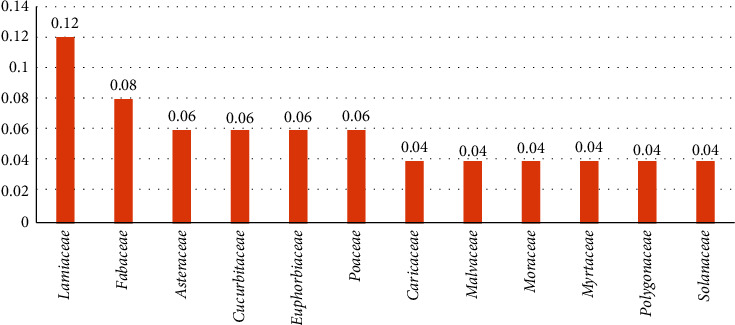
Family use values of 12 of the 28 families.

The family use value (FUV) for the 28 families ranged from 0.01 to 0.12. *Lamiaceae* was the most frequently used plant family to treat diarrhea (FUV = 0.12), followed by *Fabaceae* (FUV = 0.08), and *Asteraceae*, *Cucurbitaceae*, *Euphorbiaceae*, and *Poaceae*, all with an FUV of 0.06. Additionally, 21.4% and 57.1% of the families had FUVs of 0.04 and 0.02, respectively (Figure 3).

### 3.4. Plant Parts Used to Treat Diarrhea

**Figure 4:**
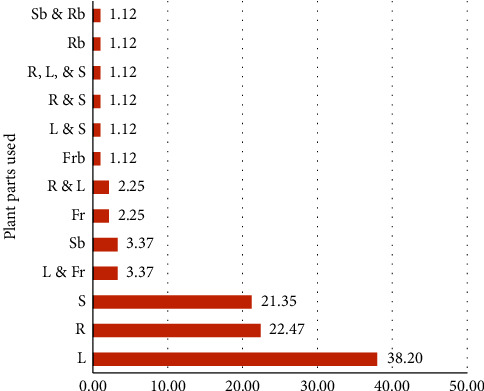
Plant parts used for the treatment of diarrhea. Note: plant parts used (Fb = fruit bark, Fr = fruit, L = Leaf, R = root, Rb = root bark, S = seed, and Sb = stem bark).

The top five plant parts used to treat diarrhea were leaves (38.2%), roots (22.47%), stems (21.35%), leaves and fruits (3.37%), and stem bark (3.37%) (Figure 4). It was followed by fruit (2.25%), root (2.25%), and leaf (2.25%), with the remaining plant parts and combinations of plant parts contributing 1.12% each to the treatment of diarrhea.

### 3.5. Gap Analysis of Whether the Traditional Claims Are Tested by *In Vitro* Trials

No *in vitro* trials have been conducted for *Acacia etbaica, Acacia abyssinica, Aloe* spp.*, Anogeissus leiocarpa, Artemisia abyssinica, Balanites aegyptiaca, Carissa spinarum, Cucumis ficifolius, Eragrostis tef, Ficus vasta, Heteromorpha arborescens, Hordeum vulgare, Justicia schimperiana, Linum usitatissimum, Malva parviflora, Mentha piperita, Momordica foetida, Gossypium barbadense, Prunus persica, Satureja punctate, Senna didymobotrya, Solanecio gigas, Solanum nigrum, Verbascum sinaiticum, Verbena officinalis,* and *Vernonia adoensis* (Table 2). Therefore, future research studies can test their effectiveness against castor oil-induced diarrhea in animal models.

However, different extracts of 18 medicinal plants traditionally used to treat diarrhea by the people in Amhara region (36%) have been proved experimentally in animal models. They are *Calpurnia aurea* [41], *Clutia abyssinica* [42], *Coffea arabica* [43], *Cordia africana* [44], *Croton macrostachyus* [45], *Ficus thonningii* [46], *Leonotis ocymifolia* [47], *Lepidium sativum* [48], *Myrtus communis* [49], *Ocimum lamiifolium* [50], *Punica granatum* [51], *Ruta chalepensis* [52], *Sorghum bicolor* [53], *Stephania abyssinica* [54], *Syzygium guineense* [55], *Withania somnifera* [56], *Zehneria scabra* [57], and *Ziziphus spina-christi* [58] (Table 2).

## 4. Discussion

Traditionally, the people in the Amhara region use different plants to treat diarrhea. In the following paragraphs, the plants used to treat diarrhea, their active components, their mechanisms of action, and, if confirmed, *in vivo* trials are discussed.

*Acacia etbaica* Schweinf and *Acacia abyssinica* may contain a variety of secondary metabolites, including alkaloids, flavonoids, tannins, saponins, anthraquinones, triterpenes, and glycosides, as shown in *Acacia etbaica* [59]. *Acacia nilotica* Willd's bark methanol extract demonstrated in vivo antidiarrheal activity against castor oil and magnesium sulfate-induced diarrhea, as well as barium chloride-induced peristalsis, using Swiss albino mice. It also exhibited *in vitro* antimicrobial activity against common diarrhea-causing microorganisms [60]. Similar effects could be attributed to *Acacia etbaica* and *Acacia abyssinica* extracts. The antidiarrheal activity of *Acacia etbaica* and *Acacia abyssinica* is likely attributed to their ability to modulate intestinal motility, preserve intestinal mucosal integrity, promote fluid absorption, activate antioxidant pathways, exert anti-inflammatory effects, demonstrate antimicrobial activity against diarrheal pathogens, suppress intestinal secretion, and modulate gut microflora.

*Aloe* spp. contains aloe emodin, aloin, aloesin, emodin, and acemannan as major active compounds [61]. Its antidiarrheal activity may be due to the anti-inflammatory, intestinal motility modulatory, antimicrobial, intestinal mucosal protection, and ion transport regulatory activities of its active components.

*Anogeissus leiocarpa* (A. Rich) Guill and Perr's bark decoction is drunk to treat diarrhea [23]. Its aqueous leaf extract significantly inhibited castor oil-induced diarrhea in rats through the inhibition of intestinal transit and reduction of the volume of the intestinal content [62]. This effect may be attributed to the activities of its components such as alkaloids, flavonoids, saponins, tannins, phenols, and glycosides [63], which act as its active constituents.

The dry or fresh leaves and roots of *Artemisia abyssinica* are ground, mixed with water, and drunk [25, 26] to treat diarrhea, and the active components of the genus, including 1,8-cineole, *β*-pinene, thujone, artemisia ketone, camphor, caryophyllene, camphene, and germacrene D [64], may contribute to its antidiarrheal activity.

The fresh leaves of *Balanites aegyptiaca* (L.) Delile are crushed, and the juice is swallowed to treat diarrhea [21]. The antidiarrheal activities of this plant may be attributed to its phytochemicals such as saponins, coumarins, triterpenes, tannins, and steroids [65].

The leaves, seeds, and fruits of *Calpurnia aurea* (Ait) Benth (Fabaceae) are applied to treat diarrhea. The antidiarrheal activity of the 80% methanol extract of this plant has been proven by its effect on castor oil-induced diarrhea in mice, which significantly reduced the time of onset of diarrhea, the frequency of defecation (total number of fecal output), and the weight of feces. The extract also showed good antimicrobial activity against all tested organisms [41]. The antidiarrheal activities of this plant may be attributed to its secondary metabolites, including alkaloids, tannins, flavonoids, saponins, steroids, and phlobatannins [66].

The roots and leaves of *Carissa spinarum* L. (*Apocynaceae*) are utilized for managing diarrhea. Its potential antidiarrheal properties may be attributed to its bioactive constituents, such as acids, glycosides, terpenoids, alkaloids, tannins, and saponins [67]. The juice of the fresh leaves of *Clutia abyssinica* Jaub and Spach is taken orally as a remedy for diarrhea. This practice is supported by an in vivo study, where its hydromethanolic root extract significantly delayed the onset of diarrhea and reduced the number of wet and total stools in a castor oil-induced diarrheal model [42]. Conversely, the crushed roots of *Clutia lanceolata* Forssk (*Euphorbiaceae*) are applied to the neck to treat diarrhea [20]. The antidiarrheal activities of this plant may be attributed to its secondary metabolites, including 5-methylcoumarins, diterpenes with a secolabdane skeleton, essential oils, alkaloids, anthraquinones, cardiac glycosides, flavonoids, phenolics, saponins, steroids, tannins, and terpenoids [68].

The dried seeds and fruits of *Coffea arabica* L. are used in coffee preparations mixed with honey, which is taken orally to treat diarrhea. The antidiarrheal activity of *C. arabica* was confirmed by an in vivo study against castor oil-induced diarrhea in Swiss albino mice [43]. Coffee's antidiarrheal effects are likely attributed to its bioactive constituents, such as chlorogenic acids and catechins [69].

The dried root bark of *Cordia africana* (*Boraginaceae*) is macerated and taken orally once daily to treat diarrhea. This practice has been supported by an in vivo study, where *C. africana* prevented castor oil-induced diarrhea and regulated intestinal motility [44]. This effect may be due to the individual, additive, or synergistic activities of its active components, such as flavonoids, alkaloids, tannins, terpenoids, saponins, steroids, anthraquinones, carbohydrates, and proteins [70].

The dry leaves of *Croton macrostachyus* De. (*Euphorbiaceae*) are powdered, mixed with water, and taken orally to treat diarrhea [34]. In the castor oil-induced model, the chloroform and methanol fractions of this plant significantly delayed diarrheal onset and decreased stool frequency and weight of feces [45]. Its activity may be due to individuals or combinations of its active components, such as flavonoids, alkaloids, tannins, saponins, terpenoids, and phenols [71].

The fresh root juice of *Cucumis ficifolius* (*Cucurbitaceae*) is taken orally to treat diarrhea. The *Cucurbitaceae* family is rich in terpenoids, glycosides, alkaloids, saponins, tannins, and steroids [72]. The antidiarrheal activities of this plant may be attributed to the mentioned secondary metabolites.

Porridge made from the seeds of *Eragrostis tef* (Zucc.) is eaten three times a day to treat diarrhea. The antidiarrheal activity of *E. tef* may be attributed to its polyphenols, including the phenolic acids p-coumaric, ferulic, protocatechuic, gentisic, vanillic, syringic, caffeic, cinnamic, and p-hydroxybenzoic, and the flavonoids apigenin, luteolin, and quercetin [73].

Chewing the dry roots of *Ficus thonningii* Blume is practiced to treat diarrhea. The antidiarrheal activity of this plant may be due to its phytochemicals such as tannins, flavonoids, saponins, and anthraquinone glycosides [46].

*Heteromorpha arborescens* (Spreng.) is utilized to treat diarrhea which may be due to its active components such as phenols, proanthocyanidins, flavonoids, alkaloids, and saponins [74]. Justicia schimperiana *(Hochst. ex Nees)* T. Anders is used for the treatment of diarrhea that may be due to its active components, such as flavonoids, alkaloids, glycosides, phenols, saponins, steroids, and terpenoids [75].

The traditional application of *Leonotis ocymifolia* for diarrhea treatment is supplemented by a study conducted in Ethiopia. According to this study, 80% methanol leaf and fruit extracts of *L. ocymifolia* reduced the frequency of wet stools, the watery content of diarrhea, and delayed the onset of diarrhea [47]. Its antidiarrheal activity may be attributed to its chemical constituents, such as phenolics, flavonoids, and alkaloids [76]. *Linum usitatissimum* L. has traditionally been used to treat diarrhea, and this may be due to its components such as methyl linolenate, methyl linoleate, *α*-linolenic acid, *α*-terpinene, terpinen-4-ol, 4-cymene, and *α*-pinene [77].

The leaves of *Malva parviflora* L. are used to treat diarrhea which may be due to its active components such as sterols, hydroxycinnamic, anthocyanins, and ferulic acid [78]. The fresh leaves of *Momordica foetida* Schumach are used to treat diarrhea. Its antidiarrheal activity may be associated with its active components, such as methyldecanoate, methyl dodecanoate, methyl tetradecanoate, methyl hexadecanoate, ethyl hexadecanoate, methyl-9-octadecenoate, methyl-8,11-octadecadienoate, methyl-9,12,15-octadecatrienoate, bis(2-ethylhexyl) phthalate, and methyl-18-methylnonadecanoate [79].

Similarly, the leaves of *Myrtus communis* L. [34] are utilized for the treatment of diarrhea. The traditional claim was confirmed by a study where the 80% methanol extract, as well as the chloroform (CF) and methanol (MF) fractions, of this plant significantly prolonged the onset of diarrhea, reduced the frequency of bowel movements, and decreased fecal output weight [49]. These antidiarrheal properties can be attributed to the presence of active components, including polyphenols, myrtucommulone, semimyrtucommulone, 1,8-cineole, *α*-pinene, myrtenyl acetate, limonene, linalool, and *α*-terpinolene [80].

The fresh leaves of *Ocimum lamiifolium* L. are used to manage diarrhea by boiling them with tea and consuming a cup of the prepared infusion. This was corroborated by an in vivo study in which the 80% methanol extract and fractions of this plant demonstrated a substantial impact on the fluid content of feces across all tested doses. Additionally, the n-butanol and distilled water fractions exhibited significant effects on the onset of diarrhea, whereas the n-hexane fraction displayed noteworthy effects on the number of wet feces, onset of diarrhea, and fluid content of feces at all tested doses [50]. The antidiarrheal activities of this plant may be attributed to its phytocomponents, such as tricyclene, bornyl acetate, *α*-pinene, *α*-terpinene, isoledene, and *β*-pinene.

The roots and leaves of *Plectranthus lactiflorus* (Vatke) Agnew are mixed with water, and the filtrate is consumed to treat diarrhea. The antidiarrheal activities of this plant may be attributed to its active components such as carvacrol, *γ*-terpinene, caryophyllene, p-cymene, trans-*α*-bergamotene, and thymoquinone [81]. The flesh fruit bark of *Punica granatum* is consumed to combat severe diarrhea [32]. Its aqueous extract displayed antidiarrheal activities against castor oil-induced diarrhea in rats [51] which may be due to its chemical components, such as hydrolyzable tannins (punicalin, punicalagin, ellagic acid, and gallic acid) and flavonoids (anthocyanins and catechins) [82].

The crushed dry roots of *Rumex nepalensis* (Spreng) are taken orally to treat diarrhea [38]. In an *in vivo* study, its hydromethanolic extract markedly delayed the onset of diarrhea and reduced the weight of wet and total feces at the test doses in a castor oil-induced diarrheal model [83]. The possible antidiarrheal activities of this plant may be related to its active components such as anthraquinones, naphthalenes, stilbenoids, flavonoids, terpenoids, phenols, and their derivatives [84].

The fresh leaves of *Ruta chalepensis* L. together with salt are chewed to treat diarrhea. Its hydromethanol (80% ME) extract prolonged the onset of diarrhea in mice and significantly reduced the frequency of stooling and weight of feces in a castor oil-induced diarrheal model [52]. The antidiarrheal activity of this medicinal plant may be ascribed to its chemical constituents such as 2-undecanone, piperonyl piperazine, 2-decalone, 2-dodecanone, decipidone, and 2-tridecanone [85]. The fresh roots of *Salvia nilotica* Jacq. are crushed and taken orally to treat diarrhea. The hydroalcoholic extract of another *Salvia* species (*S. schimperi*) exerted significant and dose-related antidiarrheal activity [86]. The antidiarrheal activity of this plant may be related to its constituents, including *β*-phellandrene, *δ*-3-carene, and caryophyllene oxide, which may have antidiarrheal properties [87].

The seed of *Senna didymobotrya* (Fresen.) is crushed, roasted, and drunk with coffee as a treatment for diarrhea. The antidiarrheal activity of this plant has not yet been tested. Its potential antidiarrheal activities may be associated with its phytocomponents, such as steroids, terpenoids, anthraquinones, tannins, saponins, glycosides, flavonoids, alkaloids, and phenols [88]. Traditionally, the leaves of *Solanecio gigas* (Vatke) C. Jeffrey are crushed and taken orally to treat diarrhea. Its antidiarrheal activity may be related to its active components such as methylene chloride, sabinene, 1-nonene, terpinen-4-ol, camphene, *γ*-terpinene, *α*-phellandrene, *β*-myrcene, 1,2,5-oxadiazol-3-carboxamide, 4,4′-azobis-2,2′-dioxide, *α*-terpinene, 1-octanamine, N-methyl, and *ρ*-cymene [89].

The dried leaves of *Solanum nigrum* L. are crushed and chewed, and the juice is swallowed to treat diarrhea. The methanol extracts of the roots and leaves of another Solanum species (*S. asterophorum* Mart) significantly and dose-relatedly inhibited the frequency of both solid and liquid stools in mice [90]. Its components, such as steroidal saponins, steroidal alkaloids, flavonoids, coumarin, lignin, organic acids, volatile oils, and polysaccharides [91], may contribute to the antidiarrheal activity of *S. asterophorum*.

Traditionally, the seed powder of *Sorghum bicolor* (Moench) is taken orally to treat diarrhea. An *in vivo* evaluation of the 80% methanol crude extract of the seeds of *this plant* in mice demonstrated inhibitory activity against castor oil-induced diarrhea, castor oil-induced enteropooling, and castor oil-induced gastrointestinal transit [53]. The antidiarrheal activity of *this plant* may be linked to its active constituents such as proteins, lipids, ash, calcium, copper, iron, zinc, gallic acid, and ferulic acids [92].

The dry roots of *Stephania abyssinica* (Dillon and A. Rich) Walp are chewed to treat diarrhea. The traditional claim was also evaluated in an *in vivo* study in mice using castor oil-induced diarrhea, which significantly prolonged the time of diarrheal induction, increased diarrhea-free time, reduced the frequency of diarrhea episodes, decreased the weight of stool, and decreased the general diarrheal score in a dose-dependent way [54]. The antidiarrheal activities of *S. abyssinica* could be attributed to the active components, including alkaloids, flavonoids, lignans, steroids, terpenoids, and coumarins [93].

*Syzygium guineense* (Willd.) DC's root or stem bark is dried, powdered, mixed with honey, and drunk orally as a treatment for diarrhea [23]. Its antidiarrheal activity may be attributed to its constituents, such as caryophyllene oxide, d-cadinene, viridiflora, epi-a-cadinol, a-cadinol, cis-calamenen-10-ol, citronellyl pentanoate, b-caryophyllene, and a-humulene [94].

The dry root of *Verbascum sinaiticum* Benth and *Verbena officinalis* L. is crushed and drunk with water to halt diarrhea. The traditional claim for the antidiarrheal activity of this plant has not yet been tested. The antidiarrheal activities of this plant may be attributed to its active compounds, such as sterols, saponins, flavonoids, phenylethanoids, and iridoid glycosides [95]. To treat diarrhea, the root decoction of *Vernonia adoensis* Sch.Bip.exWalp is taken orally [23]. Its active components phenols, saponins, flavonoids, glycosides, and tannins [96] may contribute to the antidiarrheal activities of this plant.

Fresh/dry leaves of *Withania somnifera* (L.) Dunal are crushed or squeezed and taken orally to treat diarrhea [29, 38]. In Swiss albino mice, an 80% methanol extract and solvent fractions of the leaves of this plant have been found to significantly delay the onset of diarrhea, decrease the number and weight of stools, reduce the volume and weight of intestinal contents, and decrease the motility of charcoal meal [56]. The antidiarrheal activity of this plant may be attributed to its active compounds, such as withanolides, condensed tannins, flavonoids, glycosides, free amino acids, alkaloids, steroids, volatile oils, and reducing sugars [97].

The dry leaves of *Zehneria scabra* (Linn. f.) Sond are crushed and chewed, and the juice is swallowed to stop diarrhea. The 80% methanolic leaf extract of this plant in mice resulted in a significant reduction in mean stool score, stool frequency, and fecal fluid content [57]. Its antidiarrheal activity may be due to its chemical composition, such as 3,10-dihydroxy-5,6-epoxy-*β*-ionol; 3,10-dihydroxy-5,6-epoxy-*β*-ionyl-10-O-*β*-D-glucopyranoside; cucumegastigmane I; corchoionoside C; indole-3-carboxylic acid; methyl indole-3-carboxylate; and benzyl-O-*β*-D-glucopyranoside [98]. The fresh stem bark of *Ziziphus spina-christi* (L.) Desf is taken orally as a treatment for diarrhea. Its antidiarrheal activities may be attributed to its active components such as alkaloids, sterols (*β*-sitosterol), flavonoids, triterpenoids, sapogenins, and saponins [99].

Plant secondary metabolites play their antidiarrheal roles using various mechanisms. For example, plants containing alkaloids, flavonoids, saponins, glycosides, and terpenoids modulate intestinal motility [100–104]; tannins and saponins preserve intestinal mucosal integrity [105, 106]; alkaloids, flavonoids, coumarins, glycosides, and terpenoids promote fluid absorption [101, 107, 108], flavonoids activate antioxidant pathways [109]; flavonoids, coumarins, glycosides, and terpenoids exert anti-inflammatory effects [110–114]; flavonoids, saponins, glycosides, and terpenoids demonstrate antimicrobial activity against diarrheal pathogens [115–118], tannins, saponins, and terpenoids suppress intestinal secretions [105, 119]; and terpenoids modulate gut microflora [108].

*Acacia etbaica*, *Acacia abyssinica, Anogeissus leiocarpa, Calpurnia aurea, Carissa spinarum, Clutia lanceolata, Cordia Africana, Croton macrostachyus, Cucumis ficifolius, Heteromorpha arborescens,* Justicia schimperiana, *Leonotis ocymifolia, Senna didymobotrya, Solanum nigrum, Stephania abyssinica, Withania somnifera,* and *Ziziphus spina-christi* have alkaloids as their active components. Therefore, they may modulate intestinal motility and promote fluid absorption. Alkaloids interact with opioid receptors in the gastrointestinal system, reducing bowel movement frequency [100, 120].

*Acacia etbaica, Acacia abyssinica, Anogeissus leiocarpa, Calpurnia aurea, Clutia lanceolata, Cordia Africana, Croton macrostachyus, Eragrostis tef, Ficus thonningii, Heteromorpha arborescens,* Justicia schimperiana, *Leonotis ocymifolia, Punica granatum, Rumex nepalensis, Senna didymobotrya, Solanum nigrum, Stephania abyssinica, Verbascum sinaiticum, Vernonia adoensis, Withania somnifera*, and *Ziziphus spina-christi* possess flavonoids. Consequently, their antidiarrheal activities may be achieved through the modulation of intestinal motility, promotion of fluid absorption, activation of antioxidant pathways, exertion of anti-inflammatory effects, and antimicrobial activity against diarrheal pathogens.

*Acacia etbaica, Acacia abyssinica, Anogeissus leiocarpa, Balanites aegyptiaca, Calpurnia aurea, Carissa spinarum, Clutia lanceolata, Cordia Africana, Croton macrostachyus, Cucumis ficifolius, Ficus thonningii, Heteromorpha arborescens,* Justicia schimperiana, *Senna didymobotrya, Solanum nigrum, Verbascum sinaiticum,* and *Ziziphus spina-christi* contain saponins. As a result, these plants may treat diarrhea by modulating intestinal motility, preserving intestinal mucosal integrity and antimicrobial activity against diarrheal pathogens, and suppressing intestinal secretions.

*Acacia etbaica, Acacia abyssinica, Anogeissus leiocarpa, Carissa spinarum, Clutia lanceolata, Cucumis ficifolius, Ficus thonningii,* Justicia schimperiana, *Senna didymobotrya, Verbascum sinaiticum, Vernonia adoensis,* and *Withania somnifera* have glycosides as their active components. Therefore, they may modulate intestinal motility, promote fluid absorption, exert anti-inflammatory effects, and exert antimicrobial activity against diarrheal pathogens.

*Carissa spinarum, Clutia lanceolata, Cordia Africana, Croton macrostachyus, Cucumis ficifolius,* Justicia schimperiana, *Rumex nepalensis, Senna didymobotrya, Stephania abyssinica,* and *Ziziphus spina-christi* own terpenoids in their active components. Thus, their antidiarrheal activities may be achieved through the following mechanisms: modulation of intestinal motility, promotion of fluid absorption, exertion of anti-inflammatory effects, antimicrobial activity against diarrheal pathogens, suppression of intestinal secretions, and modulation of gut microflora.

*Acacia etbaica, Acacia abyssinica, Anogeissus leiocarpa, Balanites aegyptiaca, Calpurnia aurea, Carissa spinarum, Clutia lanceolata, Cordia Africana, Croton macrostachyus, Cucumis ficifolius, Ficus thonningii, Punica granatum, Senna didymobotrya, Vernonia adoensis,* and *Withania somnifera* contain tannins in their active components. Subsequently, they treat diarrhea by preserving intestinal mucosal integrity. Tannins are known for their astringent properties, which allow them to bind and precipitate proteins. This astringency can potentially result in the reduction of inflammation and mucosal irritation. The astringent properties of tannins have been suggested as a possible mechanism underlying their antidiarrheal effects. By decreasing intestinal secretions and promoting the tightening of the intestinal mucosa, tannins may contribute to the alleviation of diarrhea [105, 121].

*Balanites aegyptiaca, Clutia lanceolata,* and *Stephania abyssinica* have coumarins as their active components. Hence, they promote fluid absorption and exert anti-inflammatory effects to halt diarrhea. Germacrene D of *Artemisia abyssinica* exhibits antimicrobial effects against diarrhea-causing pathogens [109].

The antidiarrheal activity of the chemical components of *Aloe* spp. includes polysaccharides, glycoproteins, and anthraquinones, exhibits anti-inflammatory effects [122], modulates intestinal motility [123], possesses antimicrobial activity [124], protects the intestinal mucosa [125], and regulates ion transport [126].

Essential oils in medicinal plants exhibit antimicrobial properties, targeting pathogens involved in diarrhea, while their anti-inflammatory effects can reduce gut inflammation. Furthermore, essential oils with antispasmodic activity relax smooth muscles, thereby reducing bowel spasms and the frequency of bowel movements. Some essential oils enhance fluid absorption, resulting in firmer stools and decreased diarrhea. Additionally, essential oils may have a modulating effect on the gut microbiota [127–131].

Coffee is rich in various polyphenols, such as chlorogenic acids and catechins, which possess antioxidant and anti-inflammatory properties [69]. These polyphenols demonstrate notable antidiarrheal properties through diverse mechanisms. One significant mechanism involves their antimicrobial activity [132], as well as their anti-inflammatory effects within the gastrointestinal tract, which help attenuate gut inflammation, a contributing factor to the occurrence of diarrhea. Additionally, polyphenols can modulate intestinal motility [133].

Phenols in *Croton macrostachyus, Eragrostis tef, Heteromorpha arborescens*, and *Leonotis ocymifolia* possess antimicrobial properties and can alleviate inflammation in the gastrointestinal tract, which is a contributing factor to diarrhea. They also influence intestinal motility, help mitigate toxin-induced diarrhea, and contribute to restoring the balance of fluid and electrolytes by enhancing their absorption [69, 132–135].

Certain steroids, including glucocorticoids in *Justicia schimperiana*, have been demonstrated to possess anti-inflammatory properties [136]. These properties can be advantageous in the management of conditions associated with diarrhea, such as inflammatory bowel disease. By mitigating inflammation within the gastrointestinal tract, steroids contribute to the modulation of diarrhea symptoms.

Methyl linolenate and methyl linoleate of *Linum usitatissimum* may exert their antidiarrheal action by reducing inflammation [137] in the gastrointestinal tract. Additionally, they could modulate intestinal motility [138], promoting normal bowel movements and reducing excessive stool frequency. Hydroxycinnamic acids (caffeic acid and chlorogenic acid) in *Malva parviflora* exhibit pronounced anti-inflammatory properties [139], thereby ameliorating gastrointestinal inflammation commonly associated with diarrhea.

Unsaturated fatty acid methyl esters (methyl-9-octadecenoate, methyl-8,11-octadecadienoate, and methyl-9,12,15-octadecatrienoate) in *Momordica foetida* Schumach have shown potential anti-inflammatory effects and the ability to modulate inflammatory pathways [140, 141]. These compounds thereby have the potential to ameliorate gastrointestinal inflammation commonly associated with diarrhea. Additionally, they may possess antioxidant properties [142], which can safeguard the gastrointestinal mucosa, mitigate oxidative stress, and modulate immune responses. These combined effects have the potential to contribute to the management of diarrhea.

1,8-cineole, *α*-pinene, myrtenyl acetate, limonene, linalool, and *α*-terpinolene represent volatile compounds of *Myrtus communis* [143]; tricyclene, bornyl acetate, *α*-pinene, *α*-terpinene, isoledene, and *β*-pinene in *Ocimum lamiifolium* [144]; carvacrol, *γ*-terpinene, caryophyllene, p-cymene, trans-*α*-bergamotene, and thymoquinone in *Plectranthus lactiflorus* [81] demonstrate discernible antimicrobial properties [145–149], effectively impeding the proliferation of diarrhea-causing pathogens.

Hydrolyzable tannins in *Punica granatum* (punicalin, punicalagin, ellagic acid, and gallic acid) and flavonoids (anthocyanins and catechins) have shown antimicrobial properties, potentially aiding in the elimination of bacteria, viruses, or parasites that can cause diarrhea [150, 151]. Additionally, their anti-inflammatory effects [152–154] may help alleviate inflammation in the gastrointestinal tract, which can be a contributing factor to diarrhea. Furthermore, their antioxidant activity [152, 155] could play a role by protecting the gastrointestinal mucosa from oxidative damage and helping to prevent or manage diarrhea.

Anthraquinones in *Rumex nepalensis* exert their antidiarrheal effects predominantly through the inhibition of intestinal motility [156], thereby reducing the frequency and intensity of bowel movements associated with diarrhea.

Phytochemicals in *Ruta chalepensis* such as 2-undecanone, piperonyl piperazine, 2-decalone, 2-dodecanone, decipidone, and 2-tridecanone [85] may exhibit notable antimicrobial properties, thereby exerting inhibitory effects against diarrhea-causing pathogens, encompassing bacteria, viruses, or parasites. Moreover, these compounds could modulate intestinal motility, potentially ameliorating hypermotility and reducing the frequency of bowel movements associated with diarrhea. Additionally, the presence of antispasmodic properties among these compounds might contribute to the attenuation of intestinal spasms, thereby alleviating abdominal cramping and ameliorating diarrhea-related symptoms. Furthermore, their anti-inflammatory properties could potentially play a role in mitigating inflammation within the gastrointestinal tract, consequently aiding in the management of diarrhea. Lastly, the possibility of interference with ion transport in the intestines by these compounds may influence fluid balance regulation, thus affording relief from diarrhea symptoms.

Active components of *Salvia nilotica* such as *β*-phellandrene, *δ*-3-carene, and caryophyllene may exhibit antimicrobial properties [157–159], potentially inhibiting the growth and proliferation of diarrhea-causing pathogens such as bacteria, viruses, or parasites.

Lignin in *Solanum nigrum* possesses insoluble fiber characteristics, thereby enhancing stool bulk and viscosity, which in turn promotes regular bowel movements and potentially reduces the incidence of loose stools [160]. The increased fecal bulk facilitates the expulsion of toxins and pathogens from the intestines. Moreover, lignin acts as a prebiotic [161], providing nourishment to beneficial gut bacteria that produce short-chain fatty acids (SCFAs), including butyrate. These SCFAs are pivotal in maintaining intestinal integrity and mitigating intestinal inflammation. Furthermore, lignin exhibits antioxidant properties [162], enabling it to scavenge harmful free radicals, potentially ameliorating oxidative stress in the gastrointestinal tract, and safeguarding the integrity of the intestinal mucosa.

Zinc in *Sorghum bicolor* exhibits diverse mechanisms in its potential antidiarrheal activities. It plays a vital role in preserving the integrity of the intestinal barrier by facilitating the repair of damaged intestinal epithelial cells [163] and reinforcing tight junctions, thus preventing the escape of water and electrolytes into the intestinal lumen and consequently reducing the severity and duration of diarrhea. Additionally, zinc regulates ion transport across the intestinal epithelium, curbing excessive fluid secretion by modulating the activity of specific ion channels and transporters involved in fluid secretion [164]. This restoration of ion transport balance normalizes fluid absorption and diminishes stool volume during diarrhea. Furthermore, zinc exerts immunomodulatory effects which are frequently elevated during diarrheal episodes [92]. Through the mitigation of the inflammatory response [165], zinc contributes to the resolution of diarrhea. Additionally, zinc exhibits direct antimicrobial properties [166], particularly against enteropathogens such as *Escherichia coli*, rotavirus, and *Giardia lamblia*, effectively inhibiting their proliferation and growth. Thus, zinc aids in the management of infection and alleviation of diarrhea symptoms.

Caryophyllene oxide in *Syzygium guineense* exerts potential antidiarrheal activity through various mechanisms. Its anti-inflammatory properties [167] reduce gastrointestinal tract inflammation, thereby alleviating diarrhea symptoms. Caryophyllene oxide also exhibits antimicrobial effects [159] against specific bacteria and parasites, aiding in infection control and diarrhea resolution. Additionally, its antioxidant properties [168] counteract harmful free radicals, mitigating oxidative stress and safeguarding intestinal cells, thus contributing to diarrhea management.

Withanolides in *Withania somnifera* are recognized for their anti-inflammatory and immunomodulatory properties [169], which have the potential to mitigate inflammation in the gastrointestinal tract and modulate immune responses implicated in the pathogenesis of diarrhea.

## 5. Conclusion

Many plants from the Amhara region in Ethiopia exhibited potential antidiarrheal activities, which can be attributed to their diverse secondary metabolites, including alkaloids, flavonoids, tannins, saponins, terpenoids, glycosides, and phenolics. Among the top ten cited plants, *Calpurnia aurea* contains alkaloids that interact with opioid receptors, reducing bowel movement frequency. *Verbena officinalis* contains flavonoids that modulate intestinal motility, promote fluid absorption, activate antioxidant pathways, exert anti-inflammatory effects, and exhibit antimicrobial activity against diarrheal pathogens. *Coffea arabica* is rich in polyphenols (chlorogenic acids and catechins) with antimicrobial and anti-inflammatory properties, which modulate intestinal motility and attenuate gut inflammation. *Lepidium sativum* contains terpenoids that modulate intestinal motility, promote fluid absorption, exert anti-inflammatory effects, exhibit antimicrobial activity against diarrheal pathogens, suppress intestinal secretions, and modulate gut microflora. *Artemisia abyssinica*'s germacrene D exhibits antimicrobial effects against diarrhea-causing pathogens. *Carissa spinarum* contains alkaloids, flavonoids, glycosides, and terpenoids, which may modulate intestinal motility and promote fluid absorption. *Leonotis ocymifolia* contains terpenoids that modulate intestinal motility, promote fluid absorption, exert anti-inflammatory effects, and possess antimicrobial activity against diarrheal pathogens. *Ruta chalepensis* contains 2-undecanone, piperonyl piperazine, and other compounds with antimicrobial properties, antispasmodic effects, anti-inflammatory properties, and potential interference with ion transport in the intestines. *Verbascum sinaiticum* contains flavonoids, saponins, glycosides, and terpenoids, which may modulate intestinal motility, promote fluid absorption, exert anti-inflammatory effects, and exhibit antimicrobial activity against diarrheal pathogens. *Withania somnifera*'s withanolides exhibit anti-inflammatory and immunomodulatory properties, mitigating inflammation in the gastrointestinal tract and modulating immune responses involved in diarrhea.

## Figures and Tables

**Table 1:** List of plants identified and reported to be used to treat diarrhea in the study area.

Family	Scientific name	Local name (Amh)	Plant parts used	Route of administration	Plant condition	Mode of preparation	References
Acanthaceae, just to have the column homogenized	*Justicia schimperiana (Hochst. ex Nees) T.Anders*	Smiza/Sensel	L	Oral	Dry	Smash, mix with water then drink the juice	[20]

Aloaceae	*Aloe* spp.	Eret	R	Oral	Fresh	Cutting is done to harvest the jelly juice	[21]

Apiaceae	*Heteromorpha arborescens* (Spreng). Cham. and Schitdi.	Yejib mirkuz	L	Oral	Fresh	Crush	[22]

Asteraceae	*Vernonia adoensis Sch.Bip.exWalp*	Etse mossie/mererug	R	Oral	Dry	Crashing the root and drinking the decoction	[23]
	*Artemisia abyssinica*	Chikugn	R and L	Oral	Dry	Crush and mix with water, then drink	[24]
	*Artemisia abyssinica*	Chikugn	L	Oral	Dry	The dried leaf is ground, mixed with water, and drunk	[25]
	*Artemisia abyssinica*	Chikugn	R and L	Oral	Fresh	The fresh root and leaves are crushed, mixed with water, and then drunk	[26]
	*Solanecio gigas (Vatke) C. jeffrey*	yeshikoko gomen	L	Oral	Fresh	Crush	[22]

Boraginaceae	*Cordia africana*	Wanza	Rb	Oral	Dry	Taking the maceration orally once daily until healed	[27]

Brassicaceae	*Lepidium sativum* L.	Feto	S	Oral	Dry	Seeds are ground into a paste-like food and then eaten or mixed with butter and water and then drunk	[28]
	*Lepidium sativum* L.	Feto	S	Oral	Dry	The dry seeds are pounded, powdered, and mixed with water, and the solution has to be taken orally	[29]
	*Lepidium sativum* L.	Feto	S	Oral	Dry	The seeds are crushed and mixed with milk, and then, the mixture is drunk	[26]
	*Lepidium sativum* L.	Feto	S	Oral	Dry	The dry seeds are pounded, powdered, and mixed with water, and the solution is taken orally	[30]

Caricaceae	*Carica papaya* L.	Papaya	S	Oral	Fresh	Ingest a few seeds with “Injera” for three days	[28]
	*Carissa spinarum* L.	Agam	R	Oral	Dry	The dry root is pounded, powdered, salt is added, and it is made into a solution and drunk	[29]
	*Carissa spinarum* L.	Agam	L	Oral	Dry	The leaf is powdered, mixed with *Coffea arabica* L., and drunk	[26]
	*Carissa spinarum* L.	Agam	R	Oral	Dry	The dry root is pounded, powdered, salt is added, and it is made into a solution, which is then drunk	[30]

Combretaceae	*Anogeissus leiocarpa (A. Rich) Guill. and Perr*	Kekera	Sb	Oral		Drinking the stem bark decoction	[23]

Cucurbitaceae	*Cucumis ficifolius*	Yemidir embuay	R	Oral	Fresh	The root is crushed and mixed with water before being allowed to drink	[31]
	*Zehneria scabra (Linn. f.) Sond*	Hareg eresa	L	Oral	Dry	Crush, chew, and then swallow juice	[20]
	*Momordica foetida Schumach*	Yekura hareg/Kuramechat	L	Oral	Fresh	Pound, squeeze, and then drink	[20]
	*Cucumis ficifolius*	Yemidir embuay	R	Oral	Fresh	The crushed fruits are mixed with water, and then about one liter is drunk	[32]
	*Zehneria scabra (Linn. f.) Sond*	Hareg eresa	L	Oral	Dry	Leaves are crushed and mixed with some fresh water, and then one cup of it is drunk	[33]

Euphorbiaceae	*Clutia lanceolata Forssk*	Fiyele fej	R	Dermal	Dry	It is crushed and then tied on the neck region	[20]
	*Croton macrostachyus De*	Bisana	L	Oral	Dry	Leaf powder mixed with water is taken orally	[34]
	*Clutia abyssinica Jaub. and spach*	Fiyele fej	L	Oral	Fresh	Crush	[22]

Fabaceae	*Acacia etbaica Schweinf*	Girar	R	Oral	Dry	One cup of powdered dried root with water is taken	[31]
	*Senna didymobotrya (Fresen.)*	Yeferenj digita	S	Oral	Dry/fresh	The seed is crushed and roasted, and then it is drunk with coffee	[31]
	*Calpurnia aurea (Ait). Benth*	Digita	L	Oral	Fresh	Fresh leaf soaked in water is given orally	[35]
	*Calpurnia aurea (Ait). Benth*	Digita	S	Oral	Dry	Grind and eat after pounding with honey	[20]
	*Calpurnia aurea (Ait). Benth*	Digita	L	Oral	Fresh	The fresh leaf is crushed, soaked in water for 2-3 hours, and decanted, and one glass is administered orally	[29]
	*Calpurnia aurea (Ait). Benth*	Digita	L	Oral	Fresh	The fresh leaf is crushed, soaked in water for 2-3 hours, and decanted, and one glass is administered orally	[36]
	*Acacia abyssinica*	Girar	R	Oral	Dry	The dried root is powdered and mixed with water, and one cup is drunk	[32]
	*Calpurnia aurea (Ait). Benth*	Digita	Fr	Oral	Dry	One dried and powdered pod of fruits is mixed with honey and taken before breakfast until you get relief	[32]
	*Calpurnia aurea (Ait). Benth*	Digita	L	Oral	Fresh	The fresh leaf is crushed, soaked in water for 2-3 hours, and decanted, and then one glass is administered orally	[30]
	*Calpurnia aurea (Ait). Benth*	Digita	L	Oral	Fresh	Crush and boil	[22]

Lamiaceae	*Leonotis ocymifolia*	Yeferes zeng	L and Fr	Oral	Dry	Powder of dried fruit and leaf is mixed with honey and then given	[28]
	*Leonotis ocymifolia*	Yeferes zeng	L and Fr	Oral	Dry	Dried leaf and fruit powder mixed with honey is given orally	[35]
	*Ocimum lamiifolium* L.	Damakesi	L	Oral	Fresh	Fresh leaf is boiled with tea and one cup of tea is drunk	[29]
	*Leonotis ocymifolia*	Feres zeng	L and Fr	Oral	Dry	The dried leaf and fruits are crushed, powdered, and mixed with honey, and one glass is taken orally	[36]
	*Mentha piperita* L.	Nana	L and S	Oral	Dry	Pound after mixing it with *Nigella sativa* and *A. sativum*	[21]
	*Mentha piperita* L.	Nana	L	Oral	Fresh	Pound the leaf, mix it with *A. sativum* and *R. chalepensis*, and then drink the mixture	[25]
	*Plectranthus lactiflorus (Vatke) Agnew*	Dibrk	L	Oral	Dry	The roots and leaves of *P. lactiflorus* are mixed with water, and the filtrate is drunk	[26]
	*Ocimum lamiifolium* L.	Damakesi	L	Oral	Fresh	The fresh leaves are boiled with tea, and then one cup of the mixture is drunk	[30]
	*Salvia nilotica Jacq*	Hulegeb	R	Oral	Fresh	Crush	[22]
	*Satureja punctata R.Br*	Etse-meaza/lomi kesie	R	Oral	Fresh	Use the unprocessed plant	[22]

Linaceae	*Linum usitatissimum* L	Telba	S	Oral	Dry	The powder is boiled, and then it is drunk like soup	[24]
	*Linum usitatissimum* L	Telba	S	Oral	Dry	Powder	[22]

Malvaceae	*Gossypium barbadense L*	Tite	L	Oral	Dry	Powdered and mixed with water	[37]
	*Malva parviflora* L.	Zebenya	L	Oral	Fresh	Pound	[21]

Menispermaceae	*Stephania abyssinica (Dillon and A. Rich.) Walp*	Yedimet Ain	R	Oral	Dry	Chewing	[38]

Moraceae	*Ficus vasta Forssk*	Warka	S	Oral	Bark	Dried stem bark powder with salt is given orally for cattle	[35]
	*Ficus thonningii Blume*	Chibha	R	Oral	Dry	The root is chewed	[34]
	*Ficus vasta Forssk*	Warka	Sb	Oral	Dry	The bark is crushed, powdered, mixed with salt, and given to eat	[36]

Myrtaceae	*Syzygium guineense [willd.] DC*	Dokima	Sb/Rb	Oral	Dry	Mix the powder with honey/water and then drinking	[23]
	*Myrtus communis* L.	Ades	L	Oral	Fresh	The juice of the leaf is taken orally in the morning	[34]

Poaceae	*Hordeum vulgare* L.	Gebis	S	Oral	Dry	Seeds are immersed in water and allowed to germinate before being dried, roasted, and pulverized. The powder is then heated in water and drunk till the pain subsides	[31]
	*Hordeum vulgare* L.	Tikur gebis	S	Oral	Dry	The seeds are soaked in water and made to germinate, dried, roasted, and powdered. Then the powder is boiled in water and drunk until relief is obtained	[32]
	*Eragrostis tef. (Zucc.)Trotter*	Nech teff	S	Oral	Dry	The floor porridge is eaten three times	[33]
	*Sorghum bicolor* (Moench)	Zengada	S	Oral	Dry	Powder	[22]

Polygonaceae	*Rumex nepalensis Spreng*	Yewusha milas	R	Oral	Dry	Crushing	[38]
	*Rumex abyssinicus*	Mekimeko	R	Oral	Dry	Orally take maceration once daily until the healing process is complete	[27]

Punicaceae	*Punica granatum*	Roman	Fb	Oral	Fresh	The flesh of the fruit bark is eaten continuously against heavy diarrhea	[32]

Rhamnaceae	*Ziziphus spina-christi (L.) Desf*	Geba	Sb	Oral	Fresh	Use the unprocessed plant	[22]

Rosaceae	*Prunus persica (L.) Batsch*	Kega	L	Oral	Dry	Crush, immerse in water then give	[20]

Rubiaceae	*Coffea arabica* L.	Buna	S	Oral	Dry	The powder is mixed with honey and eaten	[31]
	*Coffea arabica* L.	Buna	Fr	Oral	Dry	Grind and eat with honey	[20]
	*Coffea arabica* L.	Buna	S	Oral	Dry	The dry seed is roasted, powdered, mixed with honey, and one or two spoons are taken in the morning for three days	[29]
	*Coffea arabica* L.	Buna	S	Oral	Dry	Roast the powder and take it with honey on an empty stomach	[39]
	*Coffea arabica* L.	Buna	S	Oral	Dry	The dry seed is roasted, powdered, mixed with honey, and one or two spoons are taken in the morning for three days	[30]

Rutaceae	*Ruta chalepensis* L	Tena Adam	L	Oral	Fresh	The fresh leaf, together with salt (concoction), is chewed	[29]
	*Ruta chalepensis* L	Tena Adam	S	Oral	Dry	The pounded seed will be mixed with coffee, then drunk	[24]
	*Ruta chalepensis* L.	Tena Adam	L	Oral	Fresh	Chewing the fresh leaf together with salt (concoction)	[30]

Scrophulariaceae	*Verbascum sinaiticum Benth*	qetetina/Daba Keded	R	Oral	Dry	Crush and drink with water	[20]
	*Verbascum sinaiticum Benth*	qetetina/Daba Keded	R, L, and S	Oral	Dry	Crushing	[38]
	*Verbascum sinaiticum Benth*	qetetina/Daba Keded	R	Oral	Fresh	The juice of the root is taken orally	[34]

Solanaceae	*Solanum nigrum* L.	Awut	L	Oral	Dry	Leaves are crushed and chewed, and then the juice is swallowed	[20]
	*Withania somnifera (L.) Dunal*	Giziewa	L	Oral	Fresh/Dry	Squeezing, crushing	[29, 38]
	*Withania somnifera (L.) Dunal*	Giziewa	L	Oral	Dried or fresh	Mix the powder of 3 leaves with water and drink	[40]
	*Withania somnifera (L.) Dunal*	Giziewa	L	Oral	Fresh	The fresh leaves are crushed, squeezed, mixed with water, and then drunk	[30]

Verbenaceae	*Verbena officinalis-L*	Atuch	R	Oral	Fresh	Sap of the fresh root is chewed and swallowed for three days	[28]
	*Verbena officinalis-L*	Atuch	L	Oral	Fresh	The fresh leaves are crushed, mixed with water, and given orally	[29]
	*Verbena officinalis-L*	Atuch	R & S	Oral	Fresh	Pound the leaf, stem, and root, mix them with water, and then drink	[26]
	*Verbena officinalis-L*	Atuch	R	Oral	Fresh	Extract the root powder with water, filter it, and take the filtrate on an empty stomach	[40]
	*Verbena officinalis-L*	Atuch	L	Oral	Fresh	The fresh leaves are crushed, mixed with water, and given orally	[30]
	*Verbena officinalis-L*	Atuch	R	Oral	Fresh	Crush	[22]

Zygophyllaceae	*Balanites aegyptiaca (L.) Delile (Zygophyllaceae)*	Bedena	L	Oral	Fresh	Crush to collect juice	[21]

Note. Plant parts used (Fb = fruit bark, Fr = fruit, L = Leaf, R = root, Rb = root bark, S = seed, and Sb = stem bark).

**Table 2:** *In vivo* trials of medicinal plants to confirm the traditional claim of their utilization in treating diarrhea.

Medicinal plant	Plant parts used	Extraction method	The effects obtained	Chemical composition	Proposed mechanism of action	References for trial
*Calpurnia aurea*	Leaves	Maceration in 80% methanol	Reduced the time of diarrhea onset, defecation frequency, and fecal weight	Alkaloids, flavonoids, tannins, terpenoids, and saponins	Antimicrobial activity	[41]
*Clutia abyssinica*	Roots	Cold maceration in 80% methanol	Prolonged the onset of diarrhea, and significantly reduced the number of wet and total stools at doses of 200 and 400 mg/kg	Tannins, flavonoids, alkaloids, saponins, phenols, terpenoids, anthraquinones, and glycosides	Antisecretory effect, anti-inflammatory activity, and inhibition of peristaltic movements	[42]
*Coffea arabica*	Seeds	Cold maceration in 80% methanol	At a dosage of 400 mg/kg, there was a significant prolongation of the onset of diarrhea and a decrease in the total number of feces	Alkaloids, flavonoids, phenols, tannins, saponins, steroids, anthraquinones, glycosides, and terpenoids	Anti-inflammatory activity, increased sympathetic nerve activity, antioxidant activity, increase of the intestinal absorption of water and electrolytes	[43]
*Cordia africana*	Bark	Maceration in 80% methanol	Reduction in castor oil-induced diarrhea and intestinal fluid accumulation in a dose-dependent manner	Phenols, flavonoids, terpenoids, and saponins	Increase in water and electrolyte absorption or decrease the secretion of fluid and electrolytes, blocking the prostaglandin receptors	[44]
*Croton macrostachyus*	Leaves	Soxhlet extraction with chloroform and methanol	Delayed onset of diarrhea, reduced stool frequency, and lighter feces	Alkaloids, steroids, and terpenoids in the chloroform fraction; alkaloids, saponins, tannins, flavonoids, and cardiac glycosides in methanol fraction; and saponins, tannins, and alkaloids in aqueous fraction	Inhibition of intestinal motility and hydro-electrolytic secretion, inhibition of the intestinal secretory response induced by prostaglandins E2, promotion of fluid and electrolyte absorption	[45]
*Ficus thonningii*	Leaves	Aqueous methanolic extraction	An initial increase in purgation was observed by the 2nd hour of the test, followed by a subsequent period of constipation	Tannins, flavonoids, saponins, and anthraquinone glycosides	A dose-related reduction in intestinal motility	[46]
*Leonotis ocymifolia*	Leaves and fruits	Cold maceration in 80% methanol	Reduced diarrhea frequency, delayed onset of diarrhea, and decreased number of defecation occurrences	Alkaloids, tannins, flavonoids, and saponins	The antisecretory effect, antimotility effect, and reduction of intestinal transit	[47]
*Lepidium sativum*	Seeds	Maceration in 70% methanol	The doses of 100 and 300 mg/kg exhibited a significant antidiarrheal effect	Alkaloids, saponins, and anthraquinones	Reversing the CCh and high K + -induced contractions, dual blockade of muscarinic receptors, and Ca++ channels	[48]
*Myrtus communis*	Leaves	Maceration in 80% methanol	Significant delays of the onset of diarrhea and decreases of the frequency and weight of fecal outputs, at 200 and 400 mg/kg extract. A significant effect on the frequency and weight of wet feces, as well as the total fecal output, at 100 mg/kg of the extract	Terpenoids, flavonoids, tannins, glycosides, and saponins	Anti-inflammatory activity, suppression of the biosynthesis of eicosanoids, reduction of gastrointestinal motility	[49]
*Ocimum lamiifolium*	Leaves	Maceration in 80% methanol	The intervention resulted in a reduction in the onset of diarrhea, the number of wet feces, the weight of fresh feces, and the fluid content of feces, as well as reductions in both the volume and weight of intestinal content	Alkaloids, flavonoids, phenols, tannins, saponins, steroids, glycosides, anthraquinones, terpenoids	Anti-inflammatory activity, antioxidant activities, inhibition of prostaglandin synthesis, reduction of intestinal secretion, decrease in the synthesis of nitric oxide, and inhibition of intracellular Ca^2+^ inward current	[50]
*Punica granatum*	Peels	Aqueous extract (decoction)	Reduction in diarrhea, inhibition of wet or unformed feces production, and suppression of gastrointestinal propulsive action	Tannins, alkaloids, and flavonoids	Inhibition of intestinal motility and accumulation of intestinal fluid	[51]
*Ruta chalepensis*	Leaves	Maceration in 80% methanol	Prolonged the onset time of diarrhea and decreased the stooling frequency at 200 mg/kg and 400 mg/kg. Additionally, there was a reduction in the percentage of mean fecal output	Alkaloids, tannins, saponins, flavonoids, cardiac glycosides, terpenoids, and steroids	Inhibition of the production of prostaglandin E2 and an antispasmodic effect	[52]
*Sorghum bicolor*	Seeds	Maceration in 80% methanol	Observation of reduced intestinal fluid weight (in grams) and delayed charcoal meal propulsion through the gastrointestinal tract	Phenols, flavonoids, tannins, terpenoids, and steroids	Inhibition of motility and secretion of the gastrointestinal tract	[53]
*Stephania abyssinica*	Leaves and roots	Methanol and aqueous extract	Exhibiting an inhibitory effect on both gastrointestinal propulsion and fluid secretion, as well as demonstrating antispasmodic activity	Isoqiunol alkaloids	Decrease of hypermotility, inhibition of prostaglandin biosynthesis, anticholinergic effect, and histamine decrease	[54]
*Syzygium guineense*	Leaves	Maceration in 80% ethanol	Inhibition of intestinal propulsion, reduction in the number of watery stools, reduction of intraintestinal fluid volume, and passage of watery stool	Pentacyclic triterpenes and luteolin	Inhibition of acetylcholine-mediated intestinal smooth muscle contraction; stimulation of dopamine D2 receptor; and degradation of acetylcholinesterase	[55]
*Withania somnifera*	Leaves	Maceration in 80% methanol	Delayed the diarrhea onset at 200 and 400 mg/kg; reduced defecation of diarrheal stools (number of wet stools), total stools (wet and dry), and weight of fresh stools; decreased intraluminal fluid accumulation and charcoal meal movement	Flavonoids, alkaloids, tannins, steroids, phenols, terpenoids, and saponins	Antisecretory action, enhancing of absorption, and/or anti-motility action, anti-inflammatory activity, antispasmodic activity, calcium antagonism action	[56]
*Zehneria scabra*	Leaves	Maceration in 80% methanol	Reduced the mean stool score, wet feces, defecations, stool fluid content, intestinal motility, and weight of intestinal content	Tannins, saponins, anthraquinones, O-anthraquinones, and phenols	Inhibition of secretion, reducing intraluminal fluid accumulation, or enhancing water absorption but not delaying motility	[57]
*Ziziphus spina-christi*	Stem bark	Soxhlet extraction with methanol	Copious diarrhea was inhibited, intraluminal accumulation of fluid volume was reduced, and intestinal transit of charcoal meal decreased	Glycosides, resins, saponins, and tannins	Tannins inhibit electrolyte permeability and prostaglandin release and display antimicrobial activity. They also reduce secretion and enhance intestinal mucus resistance through protein tannate formation	[58]

## Data Availability

This published article contains all the data that were generated or analyzed during the course of this study.

## Abbreviations

CF: Chloroform fraction
DALYs: Disability-adjusted life years
ME: Methanol
MF: Methanol fraction.
